# Update on the Risks of Electronic Cigarettes—Vaping

**DOI:** 10.31486/toj.20.0012

**Published:** 2020

**Authors:** Jeremiah J. Reasoner, Betsy A. Regier, Richard Beckendorf, Russell K. McAllister

**Affiliations:** ^1^ Department of Anesthesiology, Texas A&M College of Medicine, Baylor Scott & White Medical Center, Temple, TX; ^2^ Department of Pulmonary Medicine, Texas A&M College of Medicine, Baylor Scott & White Medical Center, Temple, TX

## TO THE EDITOR

Approximately 13 years ago, electronic cigarettes (ECs), also referred to as electronic nicotine delivery systems, alternative nicotine delivery systems, e-hookahs, mods, vape pens, vaporizers, vapes, and tank systems, were introduced to the US market. ECs are lithium-ion battery–operated devices that heat a liquid, usually containing nicotine and dozens of other toxins, into an aerosol of tiny particles (sometimes called a “vapor,” although this is a misnomer) that is then inhaled. ECs rapidly gained popularity among teenagers and young adults due to marketing campaigns that touted them as a safer alternative to traditional cigarettes and as a tool for reaching the goal of smoking cessation. Because of their relatively recent introduction into the US market, the long-term health effects of ECs have not been well documented.

## PREVALENCE OF USE

Smoking tobacco has been an addictive hobby for Americans, but it peaked in the 1960s.^1^ Since then, cigarette smoking has declined. In contrast, since 2011, ECs have been gaining popularity, especially among teenagers in the 16- to 19-year-old age groups.^2^ Among adults, the prevalence of EC smoking is approximately 10%, but from 2011 to 2016, high school and middle school students self-reported a prevalence of 8% traditional cigarette usage vs 11.8% of EC usage. In middle school students, EC use increased from 0.6% in 2017 to 10.5% in 2019.^3^ In high school students, EC use increased from 11.7% in 2017 to 27.5% in 2019.^4^ According to the 2018 National Youth Tobacco Survey, 20.8% of high school students have smoked ECs at least once in the last 30 days. ECs, manufactured in the shapes of writing instruments and memory flash drives, are sleek and easily concealable, fostering surreptitious use in public venues. The US Food and Drug Administration (FDA) continues to propose and implement regulations designed to control ECs among minors and in academic settings.

## HISTORIC PERSPECTIVE

Several generations of ECs have been developed, with original concepts dating back to 1927. However, in 2003, Hon Lik, a pharmacist and inventor in Beijing, China, created the first commercially successful EC. The first generation of the Lik invention looked similar to a cigarette; however, the current fourth generation looks more like a pen, hence the name “vaping pens.”^5^

## ELECTRONIC CIGARETTES VS COMBUSTIBLE TRADITIONAL CIGARETTES

Combustible cigarettes, as the name implies, depend on the combustion of paper and tobacco to generate smoke that is inhaled into the lungs, transporting tar, nicotine, carbon monoxide, and more than 30 other chemicals. ECs rely on a heating element to aerosolize a liquid containing nicotine and assorted flavors.

## INTENTIONAL CHEMICAL ADDITIVES AND UNINTENTIONAL BYPRODUCTS OF COMBUSTION IN ELECTRONIC CIGARETTES

According to the Surgeon General's report from 2016, traditional combustible cigarettes contain more than 7,000 carcinogens and have been linked to the development of lung cancer and chronic obstructive pulmonary disease. ECs are not entirely free of these compounds and may also contain propylene glycol, glycerol, liquid flavors, heavy metals, nicotine, and volatile organic compounds (VOCs).^6,7^

Formaldehyde, a product of propylene glycol and glycerol vapor degradation, is a cancer-causing substance that may form if the EC liquid overheats or not enough liquid reaches the heating element (known as a dry puff). Formaldehyde can cause an increase in airway inflammatory markers up to 10 times more than traditional cigarettes.^6^

ECs aerosolize nicotine, and concentrations are typically between 6 to 24 mg/mL but can be as high as 100 mg/mL.^8^ Online vaping and EC stores sell a variety of EC liquids, nicotine salts, and vaping liquids containing cannabidiol (CBD) and tetrahydrocannabinol (THC). Product labels display descriptions of nicotine or CBD concentrations but do not provide information on the additional chemical ingredients used to flavor the liquids. The FDA laboratory, as well as others, has identified additional aerosolized potential toxins that are widely reported in the literature but not on the labels of the vaping products. These compounds include diacetyl, acrolein, diethylene glycol, heavy metals, and carcinogens such as acetaldehyde and benzenes.

## HEALTH EFFECTS OF ELECTRONIC CIGARETTES

Many popular flavors, such as “Pineapple Melon Swirl” and “Apple Tobacco,” are associated with inflammation and damage to the lungs. The artificial flavors “Hot Cinnamon Candies,” “Banana Pudding,” and “Menthol Tobacco” are also among the flavors that induce epithelial cell dysfunction in the lungs.^9^

VOCs such as propylene oxide, acrylamide, acrylonitrile, and crotonaldehyde can cause eye, nose, and throat irritation, headache, and nausea and can also lead to damage of the liver, kidney, and nervous system.^10^

The organic compound diacetyl, a common component of ECs, is a member of a general class known as diketones and is found in a variety of foods and drinks. Diacetyl is used for butter flavoring in foods such as microwave popcorn and potato chips and is also found in dairy products, coffee, cocoa, and alcoholic beverages. Diacetyl is approved for ingestion but has damaging effects when inhaled. It can cause a serious pulmonary disease, bronchiolitis obliterans, that was first demonstrated in workers at popcorn factories who were exposed to diacetyl emissions.^11^ Bronchiolitis obliterans is an irreversible condition characterized by fibrosis of terminal and distal bronchioles as a result of inflammation caused by chemical particles. When inhaled, diacetyl causes direct damage to the lung epithelium and a reactive inflammatory response to the subepithelial structures which concomitantly results in dysregulation of tissue repair. The damaged subepithelium induces fibroproliferation of abnormal epithelium of the small airways. Histopathologic evaluation of lung tissue damaged by bronchiolitis obliterans demonstrates damage to the distal airways. Distal bronchiole damage and fibrosis lead to peribronchial inflammation, mucus accumulation, and scarring that then lead to the obstructive pattern of lung disease.^12^

Researchers reporting in the *New England Journal of Medicine*, as well as the Centers for Disease Control and Prevention (CDC), have implicated vitamin E acetate in the development of EC, or vaping, product use–associated lung injury (EVALI). Vitamin E acetate is typically used as a thickening agent in THC-containing EC liquids. The CDC studies of lung samples from 29 patients with EVALI submitted from 10 states demonstrated that all the lung samples contained vitamin E acetate.^13^ In the 2019 study by Blount et al, health departments assigned confirmed EVALI case status for 25 patients and probable EVALI case status for 26 patients. Vitamin E acetate was identified in bronchoalveolar lavage fluid obtained from 48 of 51 case patients (94%) in 16 states but not in the fluid obtained from the healthy comparator group.^14^ The CDC and state agencies have reported 2,602 lung injury cases that required hospitalization and 59 deaths linked to vaping.^15^ The CDC recommends that no one should use vaping products containing vitamin E and THC, particularly from informal sources such as acquaintances or online dealers.

In addition to the known adverse health effects of these isolated and characterized chemicals, reactions between the substances can produce new chemicals with unknown effects on the airways and lung tissue. A study published in *Nicotine and Tobacco Research* demonstrated that the chemicals in EC liquids are unstable and reactive with the solvent propylene glycol and create new chemical compounds with their own profiles of toxicologic properties.^16^

## EXPLOSIONS, BATTERY MALFUNCTION, INJURIES

In addition to the chemical hazards of the inhaled toxins, device malfunction is another hazard. In February 2019, a 24-year-old man died after an EC exploded in his mouth and damaged his carotid artery, causing cerebral infarction and herniation.^17^ This story is just one of many about the devastating injuries caused by malfunctioning ECs. A study examining the frequency of explosions and burn injuries related to EC use found more than 2,000 cases from 2015 to 2017.^18^ ECs are powered by lithium-ion batteries that can short circuit and explode. Multiple reasons can cause the malfunction of batteries, including improper charging and lack of fail-safe mechanisms. Lack of federal regulations for EC batteries allows companies to use cheap and low-quality batteries that are not equipped with fail-safe mechanisms, resulting in an increased risk of malfunction. Consumers are generally unaware of these product differences.^19^ A systematic review of adverse events associated with ECs suggests that multiple human factors, including lack of manufacturing and design standardization and frequent consumer mishandling, have led to an increased risk of battery ignition and explosion.^20^

## SUMMARY

EC use is associated with significant morbidity and mortality from chemical lung injury and malfunctioning devices. Chemicals found in EC liquids such as diacetyl, formaldehyde, and vitamin E acetate have been implicated in severe, and sometimes fatal, lung disease. Many state legislatures are moving toward regulating the devices because of their inherent dangers. As advocates for our patients, we should actively discourage the use of these products until more information is known about how—or if—they can be used safely.
